# Personalizing mechanical ventilation according to physiologic parameters to stabilize alveoli and minimize ventilator induced lung injury (VILI)

**DOI:** 10.1186/s40635-017-0121-x

**Published:** 2017-02-02

**Authors:** Gary F. Nieman, Joshua Satalin, Penny Andrews, Hani Aiash, Nader M. Habashi, Louis A. Gatto

**Affiliations:** 1 Department of Surgery, SUNY Upstate Medical University, Syracuse, NY USA; 2 Intensive Care Online (ICON), Baltimore, MD USA; 3 Department of Trauma Critical Care Medicine, R Adams Cowley Shock Trauma Center, University of Maryland, Baltimore, MD USA; 4 Biological Sciences Department, Biological Sciences Department, SUNY Cortland, Cortland, NY USA; 5 Cardiopulmonary Critical Care Lab, Department of Surgery, Upstate Medical University, 750 East Adams Street, Syracuse, NY 13210 USA

**Keywords:** ARDS, VILI, Personalizing mechanical ventilation, Open lung ventilation, PEEP

## Abstract

It has been shown that mechanical ventilation in patients with, or at high-risk for, the development of acute respiratory distress syndrome (ARDS) can be a double-edged sword. If the mechanical breath is improperly set, it can amplify the lung injury associated with ARDS, causing a secondary ventilator-induced lung injury (VILI). Conversely, the mechanical breath can be adjusted to minimize VILI, which can reduce ARDS mortality. The current standard of care ventilation strategy to minimize VILI attempts to reduce alveolar over-distension and recruitment-derecruitment (R/D) by lowering tidal volume (Vt) to 6 cc/kg combined with adjusting positive-end expiratory pressure (PEEP) based on a sliding scale directed by changes in oxygenation. Thus, Vt is often but not always set as a “one-size-fits-all” approach and although PEEP is often set arbitrarily at 5 cmH_2_O, it may be personalized according to changes in a physiologic parameter, most often to oxygenation. However, there is evidence that oxygenation as a method to optimize PEEP is not congruent with the PEEP levels necessary to maintain an open and stable lung. Thus, optimal PEEP might not be personalized to the lung pathology of an individual patient using oxygenation as the physiologic feedback system. Multiple methods of personalizing PEEP have been tested and include dead space, lung compliance, lung stress and strain, ventilation patterns using computed tomography (CT) or electrical impedance tomography (EIT), inflection points on the pressure/volume curve (P/V), and the slope of the expiratory flow curve using airway pressure release ventilation (APRV). Although many studies have shown that personalizing PEEP is possible, there is no consensus as to the optimal technique. This review will assess various methods used to personalize PEEP, directed by physiologic parameters, necessary to adaptively adjust ventilator settings with progressive changes in lung pathophysiology.

## Review

Improvements in protective mechanical ventilation strategies have reduced mortality secondary to the acute respiratory distress syndrome (ARDS) from almost certain death (~70%) to the current mortality rate of ~40% [1] in the moderate to severe form of the disease [2]. Although some studies have shown a reduction in ARDS mortality [3], a recent review of the literature concluded that ARDS mortality rate remains unchanged and has not been reduced for almost 15 years [1, 4]. Thus, research emphasis has shifted from treating to preventing ARDS using preemptive ventilator strategies applied to the normal lung in patients at high-risk [5, 6]. Preemptive ventilator strategies, although not definitive, have been shown to reduce the complications of mechanically ventilated patients with the believed mechanism to be maintaining an open, homogeneously ventilated lung, and minimizing repetitive alveolar collapse and expansion (RACE) with each breath. However, existing preemptive strategies use the same “one-size-fits-all” approach that is currently used to treat established ARDS [7] and have not yet shown a clear reduction in ARDS incidence. Many physicians do not strictly stay with the recommended 6 cc/kg for all patients but make adjustment using their clinical knowledge to adjust Vt to better match the need of the patient. Moreover, PEEP and FiO_2_ are adjusted in reaction to changes in oxygenation, which has been shown not to correlate well with pathologic changes in lung mechanics that are known to cause ventilator-induced lung injury (VILI) [8, 9].

**Fig. 1:**
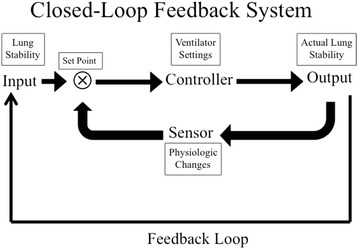
A schematic of a closed-loop feedback system that would adaptively modify ventilator settings necessary to maintain lung stability. The input is the key physiologic parameter that will be maintained by the feedback system; in this case *lung stability*. The *set point* is the parameter on the ventilator that will be adjusted to maintain the input as required. The controller is what will be adjusted to maintain the set point; in this case *ventilator setting* such as tidal volume and PEEP. The output is the desired physiologic effect; in this case *actual lung stability*. The key component of a functional feedback system is the presence of a sensor that can identify if the output is less than desirable and readjust the set point to bring the output back into compliance. *Physiologic changes* in lung function, such as oxygenation, dead space, lung compliance, infection points on the pressure/volume curve, stress index, imaging, or slope of the expiratory flow curve, can be used as the sensor to maintain the desired input

Optimization of the protective mechanical breath could be achieved if a closed-loop feedback system existed, in which the physician analyzes changes in lung physiology and uses this as feedback to adjust ventilator settings, with the goal to maintain an open and stable lung regardless of the degree of lung pathology (Fig. 1) [10, 11]. Since both alveolar opening and collapse time constants vary depending on lung injury severity and evolve as the lung pathology improves or deteriorates, ventilator settings must be constantly adjusted to fit the specific needs of the individual [12–19]. The components that comprise the Mechanical Breath Profile (MB_P_) (i.e., airway pressures, flows, volumes, rates, and the duration that they are applied during each breath) have been targeted for personalization [20–22], but personalized PEEP has been the most studied. Multiple studies have reviewed or tested methods to apply PEEP using the pathology of the lung [23–30]. However, no consensus has been reached on what that optimal strategy is that can lead to the personalization of PEEP in the protective mechanical breath.

In order to determine that strategy, the mechanism by which positive pressure ventilation injures lung tissue must first be understood. Thus, this review will discuss the current postulated mechanisms of VILI at the alveolar level. Using our understanding of the dynamic pathophysiology that occurs in the microenvironment (i.e., alveoli and alveolar ducts), we can form hypotheses on the optimal method of personalizing PEEP necessary to prevent progressive acute lung injury (ALI). Setting the ideal PEEP to stabilize the lung is an important parameter in reducing VILI and will be the focus of this review, it must be remembered that the entire MB_P_ must be adjusted properly to maximize lung protection.

### Mechanisms of VILI in the microenvironment—alveoli and alveolar ducts

**Fig. 2:**
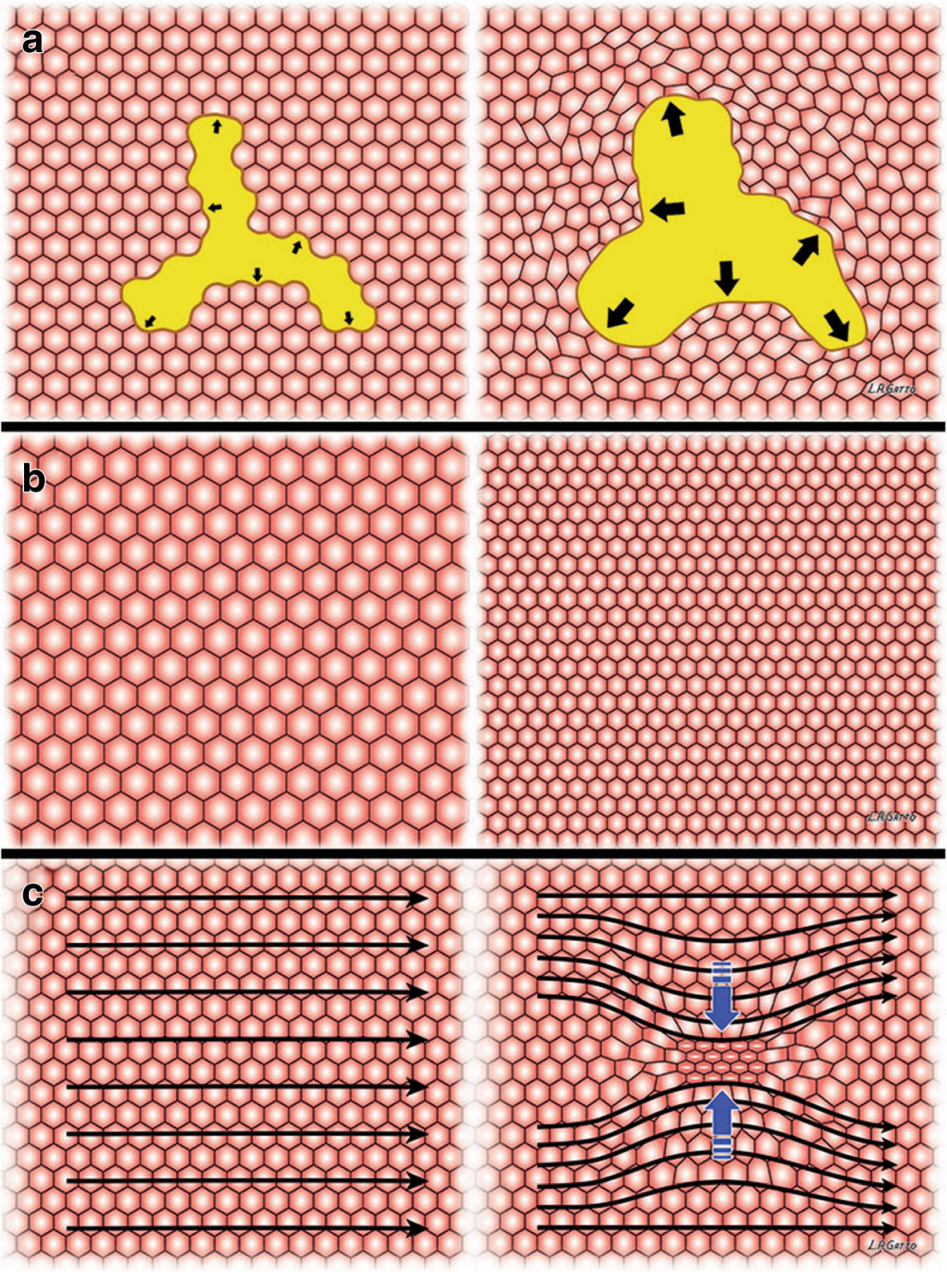
The three mechanical mechanisms of ventilator-induced lung injury (VILI) include: **a** over-distension of tissue caused by excessive volume and pressure, **b** alveolar collapse and reopening with each breath secondary to surfactant deactivation, which causes a dynamic strain-induced tissue trauma, and **c** stress-concentrators caused by heterogeneous ventilation with open alveoli adjacent to collapsed or edema-filled alveoli. **a** An alveolar duct (*yellow*) is shown surrounded by alveoli represented by hexagons. Low volume/pressure (*small arrows*) do not over-distend alveolar ducts or distort surrounding alveoli. High volume/pressure (*large arrows*) over-distend alveolar ducts and distort surrounding alveoli that can lead to stress-failure in these tissues [40]. **b** Surfactant deactivation is a hallmark of ARDS and will result in alveolar collapse at end expiration and reopening during inspiration. Following loss of surfactant function at inspiration alveoli (*hexagons*) are fully inflated. However, unless end expiratory pressure is increased alveoli collapse at expiration (*hexagons* significantly reduced in size). This alveolar recruitment/derecruitment with each breath causes severe shear stress-induced tissue trauma [116, 117]. **c** Homogeneous ventilation is represented by uniformly open alveoli (*hexagons*) and the interdependence of these alveoli with shared wall results in a very stable structure [118]. Internal force lines (*black arrows*) are uniform across the homogeneously inflated lung tissue. [119]. Heterogeneous Ventilation, where isolated areas of alveolar collapse occur (*blue arrows*) disrupts the stability of alveolar interdependence such that stress is no longer evenly distributed across the tissue. Thus, heterogeneous tissue inflation causes a significant concentration of stress in the areas surrounding the collapse. Internal force lines bow in toward the collapsed alveoli and concentrate the stress, represented by the black stress lines becoming closer together, around the area of collapse. This stress-concentration would exacerbate tissue damage in the area surrounding the collapse [33]

Although there is still debate [31], there is a great deal of literature supporting three mechanisms by which alveoli and alveolar ducts are injured during mechanical ventilation: (1) over-distension (OD) [32]; 2) dynamic recruitment and derecruitment (R/D) causing a significant dynamic strain with each breath; and (3) stress-concentration (S-C) that occurs between open and collapse or edema-filled alveoli (Fig. 2) [33, 34]. Tissue damage, secondary to these mechanical injuries, results in a secondary inflammatory injury known as biotrauma [35], which exacerbates the primary mechanical injury. However, it remains unknown which of these three mechanisms plays the greatest role in VILI pathology. This critical information is needed to determine how PEEP should be applied when attempting to block the most injurious VILI component(s). The following is a review on the relative importance of each of the above VILI mechanisms.

#### Alveolar over-distension (OD)

It is well known that ARDS causes a heterogeneous injury with collapsed or edema-filled lung adjacent to normal lung tissue. Ever since the publication of the clinical trial showing that low tidal volume (Vt) reduced ARDS mortality, the presumed mechanism for this protection was a reduction in over-distension of the normal lung tissue [7]. Gattinoni et al. reinforced this hypothesis using the term ‘Baby Lung’ for the remaining normal lung tissue in patients with ARDS. They hypothesized that the majority of the Vt would be delivered to the more compliant normal [baby] lung, thereby causing tissue injury by over-distension [32]. Most of the data supporting alveolar OD as a mechanism of VILI did not directly measure the change in alveolar size but rather the change in lung tissue density measured using computerized tomography (CT) [36]. Using CT, lung parenchyma is classified as a gas/tissue ratio in four categories: (1) not-inflated; (2) poorly inflated; (3) well-inflated; and (4) overinflated [33, 36]. Lung areas in the overinflated ‘Baby Lung’ category are hypothesized to be the tissue damaged during tidal ventilation, thus, reducing Vt would reduce tissue stretch and VILI and is believed to be the mechanism for the reduced mortality using low Vt ventilation [7].

However, a great deal of literature supports the concept that over-distension in normal lung tissue (i.e., Baby Lung) will not cause the histopathology typical of VILI, although it may cause tears in airways leading to a pneumothorax. Direct assessment of alveolar size change, using multiple techniques, have shown that alveoli do not expand significantly, as would a rubber balloon, with high volumes or pressures [37, 38]. Others have shown heterogeneous changes in individual alveolar size and shape with lung inflation but also did not show balloon-like overexpansion [39]. The site of over-distension and potential rupture may be the alveolar duct, rather than the individual alveoli (Fig. 2a) [40]. Early work by Dreyfuss et al. demonstrated that high lung volume and airway pressure sufficient to cause over-distension, induced lung damage but did not cause injury as long as dynamic alveolar strain secondary to alveolar recruitment/derecruitment (R/D) was prevented with adequate PEEP [41]. Similarly, Seah et al. showed that over-distension caused by high Vt did not cause lung histopathology unless it was combined with high dynamic strain when PEEP is set at zero [42, 43]. Using a novel method of polarized gas inhalation, which can identify the dynamic change in structures as small as alveoli and alveolar ducts, it was shown that increasing lung volume with PEEP actually decreased alveolar size, while increasing alveolar number [44]. Thus, the ‘hyper-inflated’ lung tissue seen on CT might not be caused by over-distended alveoli but rather by an increase in the number of smaller, newly recruited alveoli. In summary, the role of gross alveolar over-distension (i.e., balloon-like overexpansion) as the primary mechanism of VILI is still in question with many studies demonstrating that dynamic alveolar strain (i.e., R/D) and not OD is the primary mechanism of VILI [45, 46]. These studies are further supported in the clinically meta-analysis by Amato, which demonstrated ARDS outcome was associated with driving pressure or dynamic tidal R/D rather than static end inspiratory tidal volume/distension at given plateau pressure [22].

#### Alveolar recruitment/derecruitment (R/D)

The ability to adjust mechanical ventilator settings necessary to stabilize the lung during expiration is seen as a crucial method of reducing R/D and thus lung damage. Most studies have shown that a high static airway pressure (OD) with minimal dynamic strain (i.e., alveolar collapse and reopening) will not cause VILI [41, 42, 47, 48]. Direct measurement of alveolar R/D using in vivo microscopy demonstrated that stabilizing alveoli with adequate PEEP significantly reduced ALI [49]. The pathologic role of R/D was best evidenced in studies in which animals were ventilated at a high peak lung volume (high static strain) associated with lung over-distension with and without high dynamic strain (R/D). High static strain did not cause the histopathology and pulmonary edema characteristic of ARDS unless combined with high dynamic strain. Increasing Vt and reducing PEEP were used to cause high dynamic strain (Fig. 2b), while reducing Vt and increasing PEEP were used to cause low dynamic strain [47, 48]. Combined, these studies further demonstrate that dynamic strain caused by alveolar R/D, and not alveolar over-distension as was originally thought, is the main mechanism of VILI, which drives progressive ALI. Thus, if alveolar collapse during expiration can be prevented with properly adjusted PEEP, VILI should be dramatically reduced.

#### Alveolar stress concentrators (SC)

Recent work has identified another VILI mechanism, which occurs during heterogeneous ventilation when open alveoli are adjacent to collapsed or edema filled alveoli, which sets up stress-concentrators generating excessive strain across alveolar walls (Fig. 2c) [33, 34]. Retamal et al. demonstrated, in a novel heterogeneous rat lung injury model, that injurious stresses occur at the interface between collapsed and expanded [34]. They hypothesized that a local non-lobar atelectasis would act as a SC significantly exacerbating tissue damage in these areas. Their data supported this hypothesis, demonstrating increased inflammation and structural injury in the healthy tissue that was adjacent to the collapsed tissue during mechanical ventilation [34]. Cressoni et al. hypothesized that the mechanism of VILI in lungs with ALI was due to the presence of local inhomogeneities acting as SC [33]. The presence of local inhomogeneities was identified using CT in patients with ARDS. Increased lung inhomogeneity was correlated with the severity of ARDS and was the only variable independently associated with mortality. Increasing PEEP reduced lung inhomogeneity. Borges et al. showed increased inflammation in the lung tissue associated with lung inhomogeneities using combined positron emission tomography (PET) and CT, further supporting these studies [50]. Wellman et al. further supported the work of Borges and demonstrated that regional tidal lung strain causes local inflammation during mechanical ventilation in a sheep ARDS model [51].

The pathogenesis of ARDS can start when loss of surfactant function, caused by ventilation (either spontaneous or mechanical ventilation), leads to collapsed alveoli that act as SC in the tissue surrounding them [52, 53]. Thus, SC may be the first step in ALI pathogenesis that if unchecked will result in ARDS. It has been shown that VILI can result even with low Vt ventilation [54, 55]. It was hypothesized that the mechanism of low Vt-induced VILI was lung collapse secondary to the small ventilation volumes, resulting in heterogeneous alveolar ventilation causing SC and excessive local strain [56–58]. This hypothesis was supported by Wellman et al., who showed in early stages of ALI that: (1) high regional lung strain caused by SC may be present even when global strain is not in the pathologic range; (2) local inflammation has a positive linear relationship with tidal strain; (3) systemic inflammation (endotoxin infusion) exacerbates this inflammation; and (4) homogenizing regional tidal strain (reducing stress concentrators) by increasing PEEP and reducing Vt reduces local inflammation [51].

In summary, emerging data strongly suggests that the presence of SC is a major mechanism of VILI. The evidence also supports the hypothesis that dynamic strain caused by alveolar R/D significantly contributes to VILI pathophysiology, whereas high static strain (alveolar OD) is a less important VILI mechanism. Thus, this review will focus on how PEEP can be personalized using physiologic signals to reduce stress-concentrators (open the lung) and/or prevent dynamic strain (stabilize the lung).

### Methods and efficacy of personalizing PEEP

#### Introduction

Although the use of PEEP is the primary tool to stabilize the lung, decades of research have not discovered the optimal approach to set PEEP [59]. Multiple attempts have been made to personalize protective ventilation using changes in lung physiology. The current standard of care is a set Vt based on patient weight, while PEEP is personalized by a sliding scale based on changes in oxygenation [7]. The current methods and efficacy of personalizing PEEP to individual lung physiology used clinically will be reviewed.

#### Personalized PEEP overview

Application of PEEP, before the onset of lung injury, has prevented the development of ALI in numerous animal studies [60]. This protection was effective in multiple injury models including high endothelial permeability, high vascular pressure, high surface tension, and high airway pressure [60]. Although multiple mechanisms, including alteration of the Starling fluid flux equation (i.e., increased interstitial pressure) [61] and preservation of surfactant function [52] played a role in PEEP-induced lung protection, stabilizing alveoli is critical and has been shown to block progressive ALI [49]. These studies suggest that properly adjusted PEEP may have a significant protective effect in patients with or at high-risk of developing ARDS. However, there is currently no consensus on the optimal method to set PEEP with the goal of reducing VILI and blocking progressive ALI [62].

Caramez et al. compared the use of multiple physiologic parameters to set PEEP following a recruitment maneuver (RM) in a sheep saline lavage model [24]. They found that dynamic tidal respiratory compliance, maximum PaO_2_, maximum PaO_2_ + PaCO_2_, minimal shunt, lower inflection point (*P*_FLEX_), and the point of maximal compliance increase (Pmci,i) on the inflation limb of the pressure-volume (P-V) curve all set a similar level of PEEP. However, the PEEP obtained using the *P*_FLEX_ on the deflation limb of the P-V curve and the maximal compliance decrease on the deflation limb set a significantly higher PEEP; the true inflection point on the inflation limb and minimum PaCO_2_ set a significantly lower PEEP. They concluded that open-lung PEEP (PEEP resulting in homogenous alveolar inflation) could be identified by a decremental PEEP trial following a RM using multiple physiologic parameters (maximum dynamic tidal respiratory compliance, maximum PaO_2_, maximum PaO_2_ + PaCO_2_, minimum shunt or the inflation *P*_FLEX_, and Pmci,i).

The current standard of care uses oxygenation as the criteria to set PEEP in combination with low Vt and Pplat < 30cmH_2_O, but no difference in outcome was observed between high [63] and low PEEP [7] using this strategy. It has been shown that PEEP based on changes in oxygenation, not on changes in lung mechanics, may result in under treatment with end expiratory pressure insufficient to stabilize the lung [64]. Since oxygenation set PEEP is the current standard of care, we will begin by reviewing the evidence of efficacy for this strategy followed by other methods used to personalize PEEP that have a significant publication database for analysis.

#### PEEP personalized by oxygenation

Since the primary function of the lung is to oxygenate and ventilate, the first attempts to personalize PEEP used oxygenation to set the PEEP level. PEEP was increased with the focus on treating the blood gases until oxygenation was normalized, regardless of the impact on lung mechanics, which caused severe VILI with mortality rates between 50–75% [65]. It has been shown that oxygenation does not identify the presence of alveolar R/D (i.e., dynamic strain) [8, 9], and improved oxygenation does not always identify lung recruitment [66]. Furthermore, PEEP set to optimize oxygenation has been shown to increase lung inflammation [67]. Although, as mentioned above, there are concerns that oxygenation is not the optimal physiologic parameter by which to set protective mechanical breath parameters, it remains the current clinical standard of care [7, 68].

**Fig. 3:**
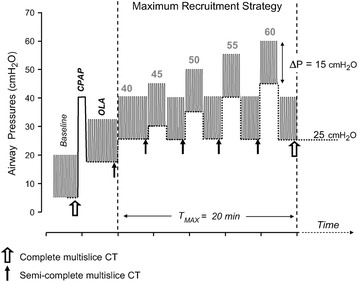
Methods used to set PEEP using a combined recruitment maneuver, and PEEP titration to a PO_2_ + PCO_2_ ≥ 400. This protocol was conducted as computed tomography (CT) was being performed to measure lung volume changes. PEEP was increased to 25 cmH_2_O with a driving pressure (∆P) of 15 cmH_2_O above PEEP. If a PO_2_ + PCO_2_ ≥ 400 was not obtained, PEEP was increased by 5 cmH_2_O for 2 min, returned to PEEP 25 cmH_2_O for 2 min and repeated until PO_2_ + PCO_2_ ≥ 400 or a PEEP of 45 cmH_2_O was obtained. *CPAP* continuous positive airway pressure, *OLA* open lung approach [70]

Chiumello et al. compared PEEP set using lung mechanics (stress index), esophageal pressure, and oxygenation and found that using oxygenation was the only method that provided PEEP levels that corresponded with lung recruitability and gradually increased with progressive lung injury [69]. This study was only designed to identify if PEEP maintained lung recruitment and thus we do not know if this strategy reduced mortality. Oxygenation may be beneficial as a physiologic feedback parameter, when used in conjunction with a RM, to identify the level of PEEP necessary to keep the newly recruited alveoli open. Borges et al. showed that following a RM, a combined PaO_2_ + PaCO_2_ > 400 mmHg identified a fully inflated lung with minimal shunt (Fig. 3) [70]. There have been three large clinical trials studying the role of PEEP in ARDS: the ALVEOLI study [63] the LOV study [71], and the ExPress study [72]. Of the three, only the ALVEOLI study used oxygenation to set PEEP, the LOV and ExPress studies used open lung ventilation and lung mechanics, respectively. Although there was no outcome difference in any of these studies, the LOV and ExPress showed a survival benefit in severe ARDS patients when treated with higher PEEP [73].

In summary, a recent review suggests that mortality has not been reduced significantly in the past 15 years (1998–2013) [4] suggesting that using other physiologic parameters to adjust mechanical ventilator settings is necessary. Although using oxygenation to set PEEP can be useful, especially when combined with a RM, the lack of direct correlation between an open and stable lung and PaO_2_ renders this personalized PEEP strategy questionable. With that said, it has been shown in a secondary analysis of the LOV and ExPress studies that patients who improved oxygenation in response to PEEP had a lower risk of death [74].

#### PEEP personalized by dead space

**Fig. 4:**
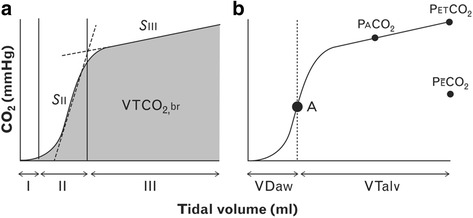
Components of volumetric capnography that can be used to personalize PEEP. **a** The three phases of capnography are: phase I contains CO_2_-free gas from the previous tidal breath; phase II (S_II_) is the steep slope contains CO_2_ from the alveolar compartment and mixed with CO_2_ in the airways from the previous breath; and phase III (S_III_) is entirely CO_2_ from alveoli and identifies the different time constants of CO_2_ being released from the capillaries and moved out of the alveoli. VTCO_2_,br is the volume of CO_2_ removed in one breath (*grey shaded area*). **b** The *black dot* (A) identifies the midpoint of S_II_ which identifies the mean airway-alveolar interface from both diffusive and convective transports. To the *left* of (A) represents airway dead space (*V*_Daw_) and to the *right* of (A) represents alveolar tidal volume (V_Talv_). PaCO_2_ = alveolar CO_2_; PETCO_2_ = end-tidal CO_2_; PECO_2_ = mixed-expired CO_2_ [77]

Another physiologic parameter that has been used to optimize PEEP is Dead Space ventilation, often expressed as the dead space (*V*_D_) to tidal volume (*V*_t_) ratio (*V*_D_/*V*t). Elevated *V*_D_/Vt is a hallmark of ARDS and has been shown to be independently associated with increased mortality [75] and has also been shown to outperform any oxygenation index parameter in predicting ARDS mortality [76]. In a review by Suarez-Sipmann, it was shown that the recent advances in volumetric capnography (VCap) make it a powerful bedside tool to assess inadequate lung protective ventilator settings and detect lung over-distension (Fig. 4) [77]. Maisch et al. used a combination of highest compliance and lowest *V*_D_/Vt to set ‘optimal’ PEEP in anesthetized patients with healthy lungs [78]. They demonstrated that this combination resulted in the maximum number of effectively recruited alveoli, and that functional residual capacity (FRC) and PaO_2_ were both insensitive at detecting over-distension. This is supported by two physiologic studies using a porcine-ARDS model that showed V_D_/Vt was useful for identifying lung collapse and the optimal PEEP necessary to maintain lung volume following a RM [79, 80]. Although using VCap or *V*_D_/Vt to set PEEP has not been tested for efficacy in a clinical trial, it is a potentially useful tool to set PEEP at the bedside.

#### PEEP personalized by imaging

**Fig. 5:**
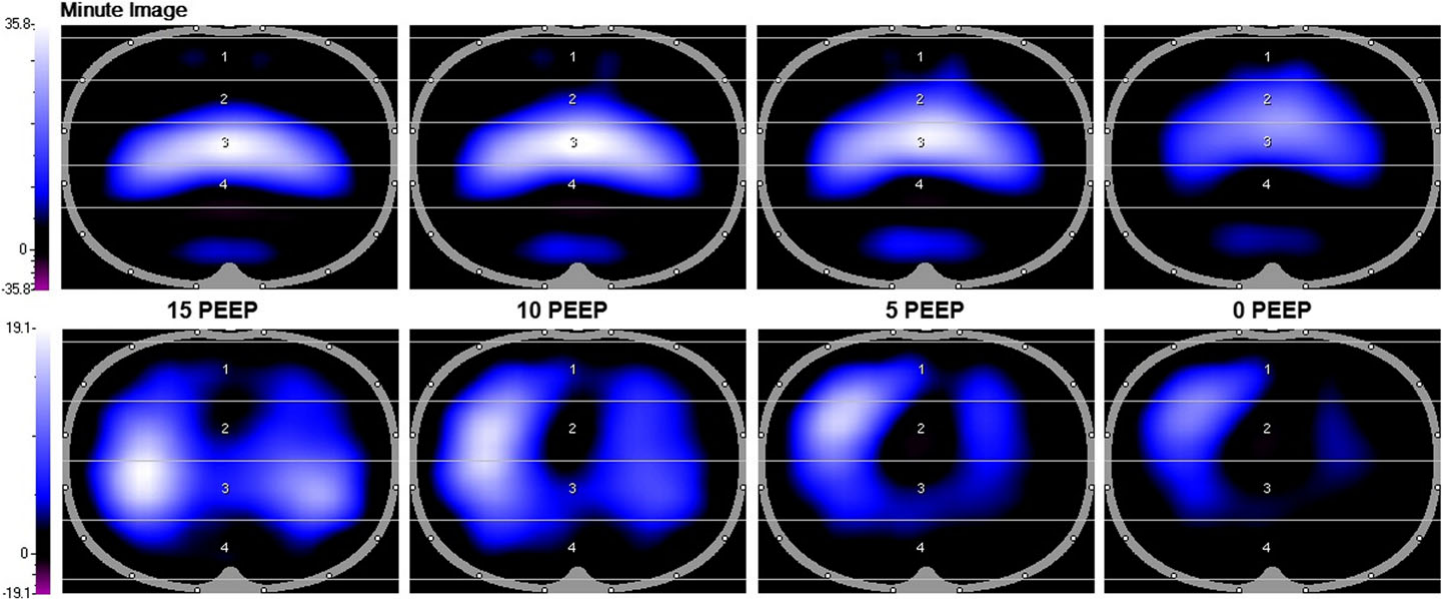
Lung ventilation during a decremental PEEP (15–0 cmH_2_O) trial measured by electrical impedance tomography (EIT) in patients following cardiac surgery. The top row images from the cranial and the bottom row images from the caudal thoracic lung level. The optimal regional compliance was different between the cranial (10 and 5 cmH_2_O) and caudal levels (15 and 10 cmH_2_O) suggesting that no single optimal PEEP may exist for all lung levels [87]

The goal of protective lung ventilation is to ‘*Open the Lung and Keep it Open’* [81] and thus, imaging should be an excellent method to identify if this goal is achieved. Indeed, CT lung scans have taught us a tremendous amount about the impact of the mechanical breath on the heterogeneous changes in lung volume during ALI [82–85]. The problem is that CT is not a tool that can be used at the bedside and thus is unavailable for treatment of most ARDS patients. However, a novel bedside device recently developed, electrical impedance tomography (EIT), allows breath-to-breath measurement of lung ventilation at the bedside. Blandkman et al. recently demonstrated that EIT and the Bohr and Enghoff calculated dead space, both identified optimal PEEP, defined as equal distribution of inspired gas volume [86]. Interestingly, they also demonstrated that *V*_D_/Vt and the normalized Slope III (SnIII) of the end tidal CO_2_ curve (Fig. 4) did not identify lung inhomogeneity. However, EIT can be used to identify the impact of PEEP on distribution of ventilation (Fig. 5) [87]. Although the majority of studies to date have simply been EIT validation experiments, a few have investigated the efficacy of EIT-guided PEEP. Muders et al., in a porcine oleic acid and abdominal hypertension-induced ARDS model, showed that EIT was effective at quantifying the amount of alveolar R/D at different PEEP levels [13]. They concluded that EIT has the ability to identify dynamic changes in tidal recruitment and thus may be an effective tool to titrate optimal PEEP. These findings were supported by Liu et al. who demonstrated that EIT can identify lung overinflation and R/D at various levels of PEEP in a porcine saline lavage-induced ARDS model [88]. Finally, Gerhard et al. compared PEEP set to ARDSnet guidelines [7] with PEEP set by EIT-derived compliance to maximize PEEP-induced lung recruitment in a porcine saline lavage ARDS model [89]. They demonstrated that EIT-guided ventilation resulted in a higher PEEP, improved global and regional compliance, improved oxygenation, and reduced lung histopathology as compared with ARDSnet protocol set PEEP. Combined data suggest that EIT may become an important tool in setting optimal PEEP.

#### PEEP personalized by lung mechanics: compliance/elastance

**Fig. 6:**
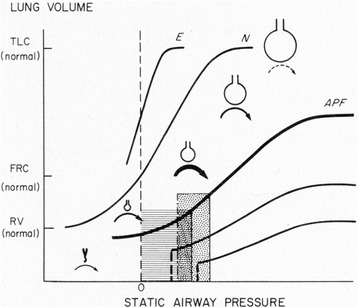
Use of the pressure/volume (*P*/*V*) curve to personalize PEEP. The shape of the *P*/*V* curve changes from normal (*N*) and differs greatly with emphysema (*E*) or acute pulmonary failure (APF). The *P*/*V* relationship during tidal ventilation is depicted in the *shaded area* with and without PEEP. *RV* regional volume at which alveoli collapse, *FRC* functional residual capacity, and *TLC* total lung capacity. Central drawing of alveoli size changes along the *P*/*V* curve [91]

Retrospective analysis of the ARMA data [68] demonstrated that lung mechanics, in the form of changes in compliance, are much more predictive of mortality than Vt [90]. Although these data are very interesting, it must be remembered that the ARMA trial was not designed to study lung mechanics and patient contribution to respiration was not identified, which may confound these interpretation of these results. The use of compliance to adjust PEEP is not a new concept. Indeed, Suter et al. first described the use of compliance adjusted PEEP over 40 years ago (Fig. 6) [91]. This early work has been supported by Chiew et al. who investigated a patient-specific, model-based, PEEP optimization strategy analyzing the relationship between the constant lung elastance (*E*_lung_) and the time-variant dynamic elastance (*E*_drs_) in ARDS patients [92]. They found that PEEP set using the model-based changes in elastance was superior to that of clinically set PEEP in maximizing lung recruitment and minimizing the work of breathing. Continuous monitoring of dynamic compliance (*C*_dyn_) as a tool to personalize PEEP was studied by Suarez-Sipmann et al. in a porcine saline lavage-induced ARDS model [93]. They compared changes in *C*_dyn_, oxygenation, and lung inflation, measured by CT following a RM plus PEEP titration trial. Initially, there was an increase in *C*_dyn_ with each reduced PEEP level. The beginning of lung collapse was defined as the PEEP level at which *C*_dyn_ began to fall. The PEEP value selected by *C*_dyn_ was compared with that selected by oxygenation and CT measurements. Both oxygenation and CT confirmed that the PEEP set by *C*_dyn_ maintained a fully open lung and concluded that *C*_dyn_ might be a valuable bedside tool to set optimal PEEP. The use of lung compliance to identify the optimally protective mechanical breath has recently been reassessed in a retrospective paper analyzing the parameters associated with increased mortality. In this statistical analysis by Amato et al., 3562 patients enrolled in nine previous ARDSnet studies were studied, and it was shown that higher plateau pressure (Pplat) was not always associated with increased mortality nor was higher PEEP always protective, whereas driving pressure (∆P = tidal volume/respiratory-system compliance) was strongly associated with survival [22]. This study reaffirms the importance of lung compliance in identifying the optimally protective mechanical breath.

#### Pressure/volume curve

**Fig. 7:**
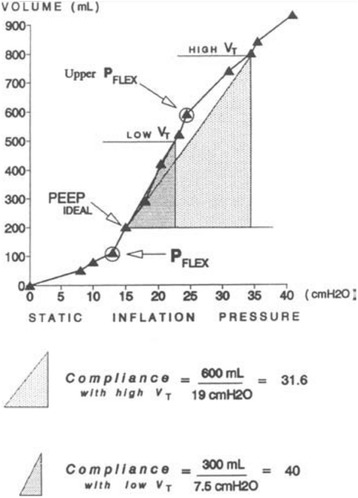
Pressure/volume (*P*/*V*) curve from an ARDS patient showing both the lower and upper inflection points (*P*_FLEX_). The hypothesis is that the lower *P*_FLEX_ is the critical alveolar opening point and the upper *P*_FLEX_ the point at which alveoli begin to over-distend, however, this hypothesis has been challenged [97, 98]. In this patient, ventilation with a high tidal volume (Vt = 10 ml/kg plus PEEP_IDEAL_ = 15 cmH_2_O) would cause over-distension since ventilation is well above the upper *P*_FLEX_. Ventilation with low Vt and PEEP_IDEAL_ was below the upper *P*_FLEX_. The calculated lung compliance was increased from 31.6 to 40 with low Vt ventilation [94]

Evidence supporting the clinical use of the whole lung pressure volume (P/V) curve as a tool to identify optimal PEEP was demonstrated by Amato et al. in two clinical trials [94, 95]. Using the P/V curve to set PEEP requires a maneuver which slowly inflates the lung, with the goal being to identify the upper and lower infection points (*P*_FLEX_) on this P/V curve (Fig. 7). The lower *P*_FLEX_ is postulated to identify the pressure at the beginning of alveolar recruitment and the upper *P*_FLEX_ is at the point of lung over-distension. They showed that a Vt of 6 cc/kg with PEEP set above the lower *P*_FLEX_ improved 28-day survival as compared with a ventilation strategy using a Vt of 12 cc/kg combined with lowest set PEEP to maintain acceptable oxygenation [95]. However, subsequent studies demonstrated that alveoli recruit continually throughout the entire inflation curve in both animal models [96, 97] and in humans. In addition, it was shown that there was a higher amount of aerated lung tissue above the point of maximum curvature on the deflation curve [98] and a higher number of alveoli above the upper *P*_FLEX_ on the deflation curve [97] as compared with the lower *P*_FLEX_ on the inflation curve. These studies suggest that using the deflation limb of the P/V curve to set PEEP may be superior, in terms of the amount of recruited lung, as compared to the inflation limb. Although using the P/V curve as a physiologic tool to personalize PEEP can be effective, generating the P/V curve is a complex procedure and there is a risk of causing hemodynamic compromise and injuring the lung during the inflation procedure. Also, ALI is always evolving and thus even if the PEEP is set properly using the P/V curve, it must be reset as the lung improves or deteriorates in function.

#### Transpulmonary pressure

Patients at risk of developing ARDS often have a decrease in chest wall compliance secondary to fluid overload and/or increased intra-abdominal pressure. Therefore, it is possible that PEEP could be set too low unless the transpulmonary pressure (Ptp) is known. Clinically, esophageal pressure (Pes) is used as a surrogate for pleural pressure (Ppl) and used to calculate Ptp. It has shown that PEEP set to maintain Ptp above 0 cmH_2_O had positive impact in both animal [99, 100] and human [101, 102] studies. Personalizing PEEP using Ptp is a physiologically sound concept since Ptp is the force that distends the lung. However, Pes is not the perfect surrogate of Ppl and thus the calculated Ptp might not be accurate. In a recent study, Huang et al. compared open-lung PEEP guided by CT to Ptp guided PEEP (i.e., PEEP set to always keep end Ptp above 0 cmH_2_O) in a porcine saline lavage lung injury model [103]. They found that Ptp-guided PEEP was unable to maintain recruited lung open with a hypothesized mechanism for this failure being an increase in the superimposed pressure between the esophageal plane and dorsal lung level. However, using Ptp to guide PEEP is a physiologically sound technique and, there is a multicenter randomized clinical trial using Ptp-guided ventilation that should help determine the efficacy of this ventilation strategy [104].

#### Stress index

**Fig. 8:**
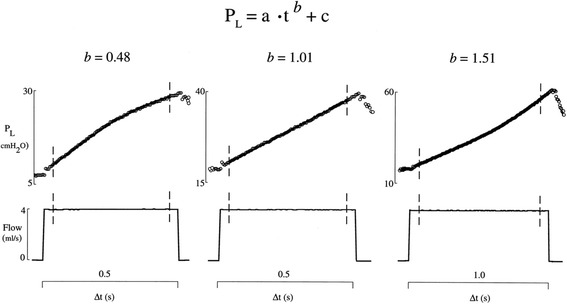
Pressure-time (*P*-*t*) curves demonstrating the concept of using stress index to personalize PEEP. Using the power equation *P*_L_ = *a* •*t* ^b^ + *c*, *b* describes the shape of the *P*-*t* curve. When *b* < 1, the shape of the curve is a downward concavity as compliance increases over time. When *b* > 1, the curve has an upward concavity as compliance decreases over time. When *b* = 1, the *P*-*t* curve is straight and compliance is constant. Adjusting tidal volume (Vt) and PEEP so that *b* = 1 produces minimal lung stress, if *b* < 1 would produce low-lung volume stress and *b* > 1 would cause high-lung volume stress [105]

In 2000, Ranieri et al. demonstrated that the pressure-time (P-t) curve generated using constant flow ventilation could be used to identify protective mechanical ventilation (Fig. 8) [105]. They used the shape of the curve to identify the stress being directed into the lung during mechanical ventilation. These studies demonstrated that if the P-t curve was straight there was minimal stress, whereas, if the curve had a downward concavity, there was increasing compliance, and if there is an upward concavity, there is decreasing compliance. The term *b* in the curve-fitting equation (Fig. 8) describes the shape of the P-t curve, and they found that a coefficient *b* of 1.00 was associated with lung protection, determined by reduced histopathology and inflammatory mediators. An extension of this work showed that a coefficient of *b* < 1 correlated with tidal recruitment, and a *b* > 1 correlated with hyperinflation, with *b* = 1 correlating with non-injurious mechanical ventilation, confirming the predictive power of this stress-index to identify injurious mechanical ventilation [26]. The accuracy of the stress-index to identify injurious mechanical ventilator settings likely to cause VILI was recently confirmed in humans [106]. CT was used to identify morphological markers of VILI including tidal hyperinflation, hyperinflated lung at expiration, and tidal recruitment. Results demonstrated that the Pplat currently considered not to cause VILI (≤30 cmH_2_O) was shown to cause tidal hyperinflation, whereas stress-index suggested a Pplat of <25 cmH_2_O (*b* < 1.05) would not and was confirmed by CT. Stress-index was also superior to Pplat at identifying the optimally protective ventilator settings in the presence of decreased chest wall compliance. Grasso et al. [107] demonstrated in ARDS patients that using stress-index to set PEEP reduced alveolar hyperinflation as compared to PEEP set using standard of care [7], although these findings have been challenged [108]. Although use of stress index to set PEEP has several physiologic advantages over using oxygenation, which is the standard of care, and there is a commercially available ventilator that can measure stress index, this technique has not been shown conclusively superior to the current strategy of adjusting PEEP using oxygenation.

#### Time controlled PEEP

**Fig. 9:**
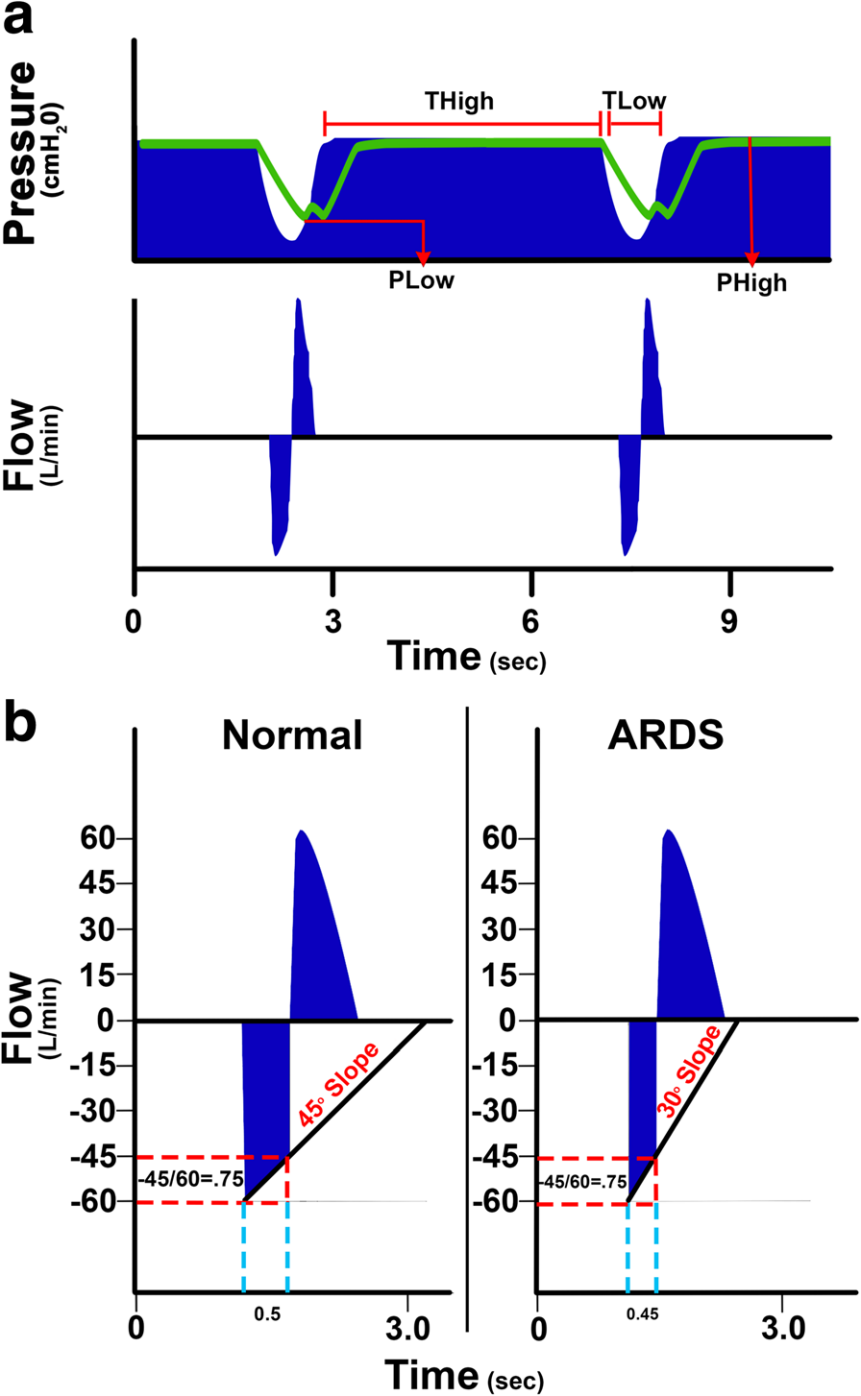
**a** Typical airway pressure release ventilation (APRV) airway *pressure* and *flow* curves. Correctly set APRV has a very brief duration at expiration (time at low pressure, *T*_Low_) and extended inspiratory duration (time at high pressure, *T*_High_) [109]. The *T*_High_ is ~90% of each breath. The two other ARPV settings are the pressure at inspiration (*P*_High_) and at expiration (*P*_Low_). *P*_High_ is set sufficiently high to recruit and open alveoli and *P*_Low_ is always set at 0 cmH_2_O to facilitate expiratory flow. However, *T*_Low_ is sufficiently short such that end-expiratory pressure (*P*_Low_) never reaches 0 cmH_2_O identified by the tracheal pressure (*green line*) maintaining a level of PEEP. **b** This figure summarizes our novel method to maintain alveolar stability by adaptively adjusting the expiratory duration as directed by the expiratory flow curve. The rate of lung collapse is seen in the normal (slope 45°) and acutely injured lung (ARDS, slope 30°). ARDS causes a more rapid lung collapse due to decreased lung compliance. Our preliminary studies have shown that if the ratio of the peak expiratory flow (*PEF*, −60 L/min) to when we end expiratory flow (*EEF*, −45 L/min) (EEF/PEF) is equal to 75% that this expiratory duration (0.5 s) is sufficient to stabilize alveoli [40, 111]. The lung with ARDS collapses more rapidly such that the EEF/PEF-75% identifies an expiratory duration of 0.45 s necessary to stabilize alveoli. Although the EEF/PEF is fixed, the expiratory duration is not, but rather adaptive and will stabilize alveoli regardless of lung injury severity. Thus, this method of setting expiratory duration is adaptive to changes in lung pathophysiology and personalizes the mechanical breath to each individual patient

A novel physiologic tool to set PEEP is the slope of the expiratory flow curve (Sl_EFC_) in conjunction with airway pressure release ventilation (APRV) (Fig. 9) [109, 110]. Although this method is not widely utilized throughout the country, it is intensely used in some hospitals and can be used as a primary mode of ventilation [109]. This time-controlled PEEP is not directly set but rather the time during expiration (*T*_LOW_) is sufficiently brief to prevent the lung from fully emptying. Thus, both lung volume and pressure (i.e., PEEP) remain at the beginning of lung reinflated. The shorter the duration of expiration, the higher the retained-end expiratory lung volume and positive-end release pressure (PERP) that remains in the lung.

It is important to understand that the personalized APRV (P-APRV) used to set time-controlled PEEP [109, 110] and inverse inspiratory:expiratory (I:E) ratio are not at all the same mechanical breath. Although there are many differences between P-APRV and inverse I:E, I will focus on the large difference in the time at expiration between these two ventilation strategies. It was shown by Neumann et al. that the ARDS lung collapses very rapidly, their data showing collapse in 0.6 s after the initiation of expiration [17]. Our work using direct observation of alveoli during mechanical ventilation has also shown a very rapid alveolar collapse in the ARDS lung [40, 111–113]. Unlike APRV, inverse ratio ventilation does not allow direct and independent adjustments of the expiratory and inspiratory times. Thus, if the expiratory duration with inverse I:E is not less than 0.4–0.6 s, which is most often the case, alveoli would have sufficient time to collapse with each breath and inverse I:E would not be defined as time controlled PEEP, since the lung was allowed time to empty.

An example of how the Sl_EFC_ will change with progressive ALI and how we can use the Sl_EFC_ to set the expiratory duration necessary to stabilize the lung is as follows: a hypothetical normal lung has been given a Sl_EFC_ of ~45° and an ARDS lung a Sl_EFC_ of ~30° (Fig. 9b). To set the optimal expiratory duration necessary to stabilize the lung, the end expiratory flow to peak expiratory flow ratio (EEF/PEF) is used. It has been shown that the ratio that best stabilizes the lung but is still able to adequately ventilate the patient or animal is 75% [40, 111, 113, 114]. In the example, the PEF is −60 L/min so to determine when to stop expiration take −60 × 0.75 = 45 L/min, and thus the clinician would terminate exhalation and reapply the continuous positive airway pressure (CPAP) at −45 L/min. In the normal lung, the expiratory duration would be 0.5 s (Fig. 9b). With the development of ALI or ARDS, the lung becomes noncompliant and collapses very rapidly decreasing the Sl_EFC_ to ~30°. Using the same equation used in the normal lung, but with a steeper slope, we see that the expiratory time has been reduced from 0.5 to 0.45 s in order to prevent alveolar collapse in this noncompliant lung (Fig. 9b). This method results in a time-controlled PEEP, effectively minimizing dynamic strain (Fig. 9b) by stabilizing alveoli that uses two mechanisms: *time* and *pressure* [110].

Multiple studies have shown that this combined method of PEEP plus a brief release time is very effective at stabilizing alveoli and alveolar ducts, reducing tissue strain [40, 111], blocking progressive ALI, and reducing ARDS incidence in a clinically applicable, high-fidelity, porcine model of sepsis and gut ischemia/reperfusion-induced ARDS [113], and in a trauma patient statistical analysis [114]. More clinical studies are necessary to confirm the efficacy of this novel method to stabilize the lung.

## Conclusions

It is clear that a high level of dynamic strain caused by alveolar R/D is a major mechanism of lung tissue damage associated with VILI. Application of PEEP is currently the primary strategy by which to minimize dynamic strain for established ARDS. In addition, early PEEP application has been effective at reducing the complication associated with mechanical ventilation in both animal and human studies. It is also clear that in order for PEEP to be effective, it must be personalized to the specific pathology of each patient’s lung. The continued high mortality rate of ARDS supports the hypothesis that the current PEEP strategies are not always effective [1]. Multiple methods to personalize PEEP have been tested and have been shown to be capable of stabilizing the lung. A large body of literature supports the use of RMs to open the lung, prior to the application of PEEP, which is set based on physiologic feedback. Recent clinical studies are attempting to personalize PEEP following RMs in humans [67, 115]. The main problem with the use of RMs is that they cannot be given very often, due to potential serious side effects, and the acutely injured lung is constantly changing. Thus, if lung pathology increases following the initial PEEP setting, lung instability would go unrecognized, causing additional VILI-induced lung damage. A novel method of personalizing PEEP is the using of expiratory flow curve during APRV. The advantage is that a RM is not required so that adjustments in expiratory duration are adaptive with progressive changes in acute lung injury, regardless if these changes are for the better or worse. Also, this method does not directly set PEEP but rather uses a short expiratory duration to generate intrinsic PEEP, which is used as a tool to stabilize the lung. Both animal studies and a human statistical analysis suggest that adjusting the release time on a breath-to-breath basis may be the optimal mechanism to adaptively personalize PEEP.

## Abbreviations

ALI: Acute lung injury
APRV: Airway pressure release ventilation
ARDS: Acute respiratory distress syndrome
CPAP: Continuous positive airway pressure
CT: Computed tomography
EEF/PEF: End expiratory flow to peak expiratory flow ratio
EIT: Electrical impedance tomography
FRC: Functional residual capacity
OD: Over-distension
P/V: Pressure/volume
P-APRV: Personalized airway pressure release ventilation
PEEP: Positive-end expiratory pressure
Pes: Esophageal pressure
*P*_FLEX_: Lower inflection point
Pmci,I: Maximal compliance increase
Ppl: Pleural pressure
Ptp: Transpulmonary pressure
R/D: Recruitment and derecruitment
RACE: Rapid alveolar collapse and expansion
RM: Recruitment maneuver
S-C: Stress concentration
SC: Stress concentrators
Sl_EFC_: Slope of the expiratory flow curve
VCap: Volumetric capnography
VILI: Ventilator-induced lung injury
Vt: Tidal volume
